# At Limits of Life: Multidisciplinary Insights Reveal Environmental Constraints on Biotic Diversity in Continental Antarctica

**DOI:** 10.1371/journal.pone.0044578

**Published:** 2012-09-19

**Authors:** Catarina Magalhães, Mark I. Stevens, S. Craig Cary, Becky A. Ball, Bryan C. Storey, Diana H. Wall, Roman Türk, Ulrike Ruprecht

**Affiliations:** 1 Centre of Marine and Environmental Research, University of Porto, Portugal, Rua dos Bragas, Porto, Portugal; 2 South Australian Museum, School of Earth and Environmental Sciences, University of Adelaide, Australia; 3 Department of Biological Sciences, University of Waikato, Hamilton, New Zealand; 4 Division of Mathematical and Natural Sciences, Arizona State University at the West Campus, Glendale, Arizona, United States of America; 5 Gateway Antarctica, University of Canterbury, Christchurch, New Zealand; 6 Department of Biology and Natural Resource Ecology Laboratory, Colorado State University, Fort Collins, Colorado, United States of America; 7 Department of Organismic Biology, University of Salzburg, Hellbrunnerstr, Salzburg, Austria; 8 College of Earth, Ocean and Environment, University of Delaware, Lewes, Delaware, United States of America; Institute of Botany, Czech Academy of Sciences, Czech Republic

## Abstract

Multitrophic communities that maintain the functionality of the extreme Antarctic terrestrial ecosystems, while the simplest of any natural community, are still challenging our knowledge about the limits to life on earth. In this study, we describe and interpret the linkage between the diversity of different trophic level communities to the geological morphology and soil geochemistry in the remote Transantarctic Mountains (Darwin Mountains, 80°S). We examined the distribution and diversity of biota (bacteria, cyanobacteria, lichens, algae, invertebrates) with respect to elevation, age of glacial drift sheets, and soil physicochemistry. Results showed an abiotic spatial gradient with respect to the diversity of the organisms across different trophic levels. More complex communities, in terms of trophic level diversity, were related to the weakly developed younger drifts (Hatherton and Britannia) with higher soil C/N ratio and lower total soluble salts content (thus lower conductivity). Our results indicate that an increase of ion concentration from younger to older drift regions drives a succession of complex to more simple communities, in terms of number of trophic levels and diversity within each group of organisms analysed. This study revealed that integrating diversity across multi-trophic levels of biotic communities with abiotic spatial heterogeneity and geological history is fundamental to understand environmental constraints influencing biological distribution in Antarctic soil ecosystems.

## Introduction

The evolutionary and biogeographic history of the Antarctic cold-desert biota reveals many components of ancient origin [1], [2]. Long-term isolation of this biota implies persistence through multiple glacial cycles [3], [4]. However, few have attempted to resolve the critical requirements for life that maintain the southern most functioning terrestrial ecosystems with the simplest and lowest diversity food web of any natural community. Organisms that survive in these extremely cold and arid Antarctic terrestrial ecosystems are subject to more environmental stresses than any other desert on the planet; dramatic physical and chemical gradients combined with extreme conditions including low temperatures, low available water and humidity, abundant freeze-thaw cycles, high salinity, low carbon and nutrient concentrations and high ultra-violet radiation [5], [6], [7], [8], [9].

Continental Antarctic soils are usually described as biologically depauperate and very simple in terms of biological diversity and food webs, since it is usually accepted that as the environmental constraints increase, fewer organisms possess the necessary adaptations [8], [9]. Faunal terrestrial communities of continental Antarctic ecosystems consist largely of simple communities of invertebrates: springtails, mites, nematodes, rotifers and tardigrades [12]. Only the vegetation forming organism, such as algae, lichen and moss occur at these extreme conditions [12], [13]. Microbial communities in Antarctic soils have received comparatively less attention in this respect, as it was previously suggested that these extreme ecosystems exhibit low diversity and abundance [14], [15]. However, contrary to earlier assumptions, recent studies based on culture-independent genetic tools are now discovering that these ecosystems contain highly diverse microbial communities [7], [16], [17], [18], [19], [20]. The trophic simplicity of Antarctic ecosystems offers a great and unique opportunity to address questions related to biodiversity, trophic relationships, succession and ecosystem functionality, and ultimately the constraints to each of these elements [7], [12].

The distribution and abundance of the Antarctic biota are subject to high spatial patterning due to the extreme heterogeneity of biogeochemical properties and climate gradients [6], [16], causing important selection pressures on micro and macrobiota distribution [6], [16], [21], [22], [23], [24]. Thus, knowing which environmental factors drive the distribution of species at different trophic levels is essential to understand ecosystem dynamics of polar terrestrial environments [16]. Studies on the environmental factors that drive habitat suitability for multitrophic community establishment, for example in the McMurdo Dry Valleys, have revealed that soil chemistry is a primary driver for establishment of soil biota [6], [16], [21], [22], [23]. Other studies have suggested that the source and composition of organic matter, availability of liquid water, and soil salinity impose strong limitations over biological colonization [23], [25], [26], [27].

Previous research on micro and macro-biotic distribution has been conducted in Antarctic extreme cold desert environments, mainly in the Victoria Land region [6], [7], [12], [18], [19], [28], [29], but it has not undertaken the level of integration across disciplines necessary to answer ecosystem-wide questions. Here, we hypothesized that abiotic characteristics, such as terrain age, glaciation history and soil geochemistry, are the main drivers of distribution and succession of multi-trophic biotic communities (bacteria, cyanobacteria, invertebrates, lichens and algae). Such a hypothesis is achievable in a landscape where the drift age of terrain and glacial advance and retreat are the major dictators of ecosystem presence and absence; one of the very few regions on earth where such a study is possible is the ice-free regions of the Darwin Mountains, Transantarctic Mountains. This work represents the first to integrate a wide multi-disciplinary dataset from around 80°S in the Darwin Mountains, Antarctica.

## Results

### Soil Characterization

**Figure 1:**
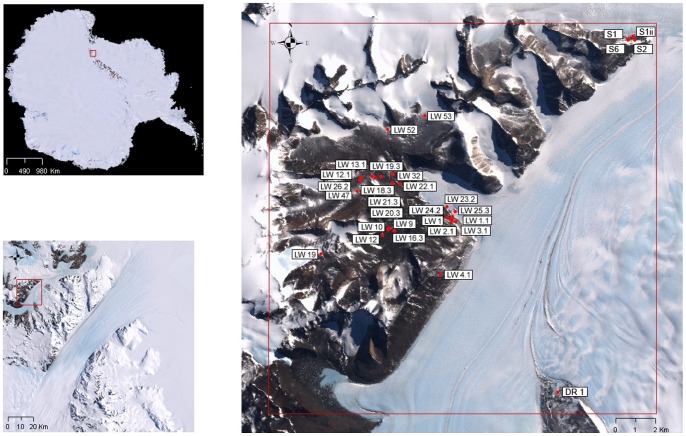
Map of Darwin-Hatherton Glacier region. Sampling sites were located around Lake Wellman (LW samples), Junction Spur (S samples) and on Dusky Ridge (DR1 sample).

**Figure 2:**
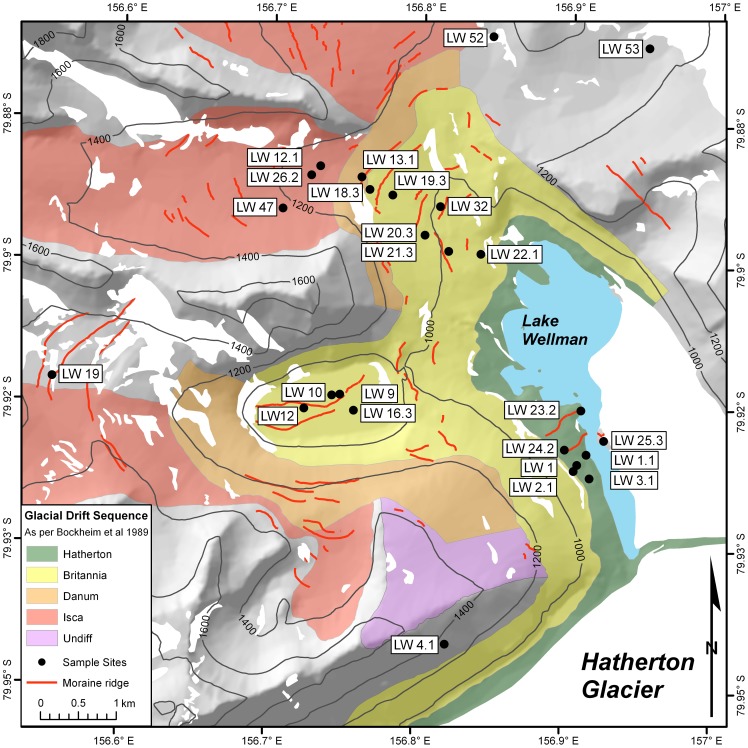
Geomorphological map of Lake Wellman area. Lake Wellman (LW) sampling sites were projected on the main drift ages modified after Bockheim et al. [30].

Soil samples collected in the ice-free regions of the Darwin Mountains (Fig. 1) were distributed in glacial drift sheets (deposits left by receding ice) ranging in age from Holocene to early Quaternary [30], [31] (Table 1, Fig. 2). From correlations with glacial deposits near McMurdo Sound and from local ^14^C dates of algae samples, Bockheim et al. [31] assigned an early Holocene age (5–6 kyr; 1 kyr = 1000 years) to the youngest Hatherton drift, an age of 10–12 kyr to the older Britannia drift, an age of circa 150 kyr to the Danum drift with the oldest Isca drift undated (Fig. 2). Ages of the drift sequences (and potential uncertainties) have been recently refined based on cosmogenic exposure ages [31] as follows: Hatherton 1 kyr, Britannia 30–40 kyr, Danum 150 kyr and an age of approx 2 million years for the oldest Isca drift. The drift sheets have different glacial morphologies, weathering and soil characteristics [30], [31]. Soils analyzed in this study showed a broad range of chemical and physical characteristics (Table 1; Table S1), and in the majority of the samples Cl, Na, Mg, NO_2_^−^ + NO_3_^−^ and Ca ions dominated (Table S1), representing the major contributors for the conductivity values (*R* ^2^ between conductivity and these ions ranged from 0.75 for Ca to 0.92 for NO_2_^−^ + NO_3_^−^, *p*<0.001). Soils were generally deficient of carbon and nitrogen (Table 1), with total nitrogen being dominated by the inorganic fraction NO_2_^−^ + NO_3_^−^ (%TN were significantly linearly related with NO_3_^−^ + NO_2_^−^ soil concentrations; R^2^ = 0.93, *p*<0.001). Two-dimensional principal components analysis (PCA) was applied to the environmental variables (Table 1; Table S1; Fig. S1) and results indicate that all samples from the Junction Spur sites (S sites) and five Lake Wellman sites (LW23.2, LW24.2, LW22.2, LW25.3, LW19) were distinguished from others by being associated with lower concentrations of NO_2_^−^ + NO_3_^−^, Cl, Mg, Ca, Na, and thus lower conductivity values (Fig. S1a) and higher C/N ratios (Fig. S1b).

**Table 1:** Geochemical properties of soil samples from all sampling sites (n.a. = not available).

	Moisture	TN	TC	OC	pH	TC/TN	Cond.	Altitude	Drift ages (a)
ID	% (g/g)	mg/g soil					µS/cm	(m)	
**LW23.2**	0.8	0.01	0.50	0.22	8.69	41.72	124.6	850	Hatherton
**LW25.3**	0.7	0.11	0.67	0.27	8.19	5.89	166.4	852	Hatherton
**LW1.1**	n.a.	n.a.	n.a.	n.a.	n.a.	n.a.	n.a.	852	Hatherton
**LW2.1**	n.a.	n.a.	n.a.	n.a.	n.a.	n.a.	n.a.	885	Britannia
**LW1**	3.8	3.51	0.95	0.31	7.52	0.27	10368.9	874	Hatherton
**LW24.2**	1.1	0.08	0.71	0.14	8.45	9.02	282.8	892	Hatherton
**LW22.1**	1.2	0.32	1.52	0.50	7.93	4.76	11.5	929	Britannia
**LW10**	4.4	2.88	0.73	0.31	8.00	0.26	7900.0	939	Britannia
**LW9**	1.9	0.75	0.21	0.18	8.03	0.28	2260.2	940	Britannia
**LW16.3**	2.2	0.74	1.117	0.59	7.61	1.59	2474.4	941	Britannia
**LW3.1**	n.a.	n.a.	n.a.	n.a.	n.a.	n.a.	n.a.	944	Hatherton
**LW12**	4.5	3.99	1.73	0.59	7.73	0.43	7915.0	955	Britannia
**LW21.3**	0.9	0.27	0.28	0.26	7.91	1.04	910.5	987	Britannia
**LW32**	11.3	3.02	1.19	0.31	8.32	0.40	9394.4	993	Britannia
**LW20.3**	0.9	0.73	0.15	0.06	7.75	0.21	1847.8	1025	Britannia
**LW19.3**	1.8	1.40	0.18	0.11	7.8	0.15	4316.8	1073	Britannia
**LW18.3**	1.4	1.31	0.39	0.15	7.94	0.30	2967.1	1101	Britannia
**LW13.1**	1.9	1.19	0.31	0.09	7.83	0.26	4381.1	1104	Danum
**LW53**	5.6	2.54	0.39	0.30	7.53	0.16	5409.4	1147	Isca
**LW52**	4.1	1.18	0.20	0.14	7.93	0.17	4628.5	1148	Isca
**LW12.1**	2.0	1.48	0.16	0.20	7.63	0.11	3492.0	1150	Isca
**LW26.2**	1.7	1.51	0.14	0.15	7.75	0.09	3603.5	1161	Isca
**LW47**	3.4	0.41	0.24	0.18	7.97	0.58	2057.7	1230	Isca
**LW19**	2.9	0.08	0.50	0.53	7.80	6.56	280.0	1371	Isca
**LW4.1**	n.a.	n.a.	n.a.	n.a.	n.a.	n.a.	n.a.	1501	Isca
**S1**	3.3	0.04	0.27	0.28	8.33	7.23	29.7	908	Hatherton
**S1ii**	2.3	0.02	0.33	0.37	7.92	17.66	12.5	927	Hatherton
**S2**	6.3	0.00	0.14	0.12	7.87	34.84	75.1	910	Hatherton
**S6**	6.4	0.16	1.38	0.85	8.92	8.78	162.7	845	Hatherton
**DR1**	2.2	1.24	14.83	14.29	7.34	11.95	1097.9	968	Hatherton

(a) Drift ages modified after Bockheim et al. [30].

### Biological Diversity

DNA profiling of the bacterial and cyanobacterial communities was performed by automated rRNA intergenic spacer analysis fragment lengths (ARISA-AFLs) and showed the presence of bacteria ARISA-AFLs in all but three samples (LW1, LW52, LW53) and the presence of cyanobacteria ARISA-AFLs in only 17 of 30 samples (Table S1). A total of 123 different ARISA-AFLs for bacteria and 68 ARISA-AFLs for cyanobacteria were identified in all samples analyzed. The highest bacterial and cyanobacterial diversity (average peak number) was observed at the Junction Spur sites. Fewer or no cyanobacteria ARISA-AFLs were registered at sites for which lower bacterial ARISA-AFLs were observed (Table S2). Indeed, the number of bacterial and cyanobacterial ARISA-AFLs for all samples were found to be linearly related (*R* ^2^ = 0.56, *p*<0.001, n = 30). Cluster patterns of the Hierarchical Cluster (HC) analysis based on cyanobacteria ARISA-AFLs profiles showed that all samples from Junction Spur and LW19 from Lake Wellman formed a distinct cluster (Fig. S2a). Cyanobacteria in the other samples taken around Lake Wellman were distributed within the remaining two clusters (Fig. S2a). In terms of bacterial community assemblage, HC analysis showed that samples LW9, LW18.3 and LW32 had very different bacterial assemblages compared to the remaining samples (Fig. S2b). Similarly to cyanobacteria, the bacterial composition from Junction Spur sites and LW19 also grouped in the same cluster (Fig. S2b).

Overall, macro-flora was found to be sparse in the Darwin Mountains. No bryophytes were observed and lichen diversity was low; with *Lecidea cacriformis* being the most widely distributed lichen in the Lake Wellman (LW16.3, LW19, LW19.3) and Junction Spur sites (Table S2). At Junction Spur a more diverse flora was found, with three more lichen species identified (*Buellia frigida*, *Acarospora gwynii* and *Lecanora fuscobrunnea*). At Junction Spur sites we also found poorly developed thalli of *Acarospora gwynnii* on the lower surface of sandstone, and these were the only sites where we found terrestrial algae, identified as Chlorophytan and Xanthophycean.

The trend of very low lichen and algae species diversity, and total absence of any bryophyte was mirrored by the faunal species diversity (Table S2). Rotifers and tardigrades were the only invertebrates found in the Lake Wellman region, and were each found at only two sites (LW19 and LW19.3, respectively; Table S2). Mites, nematodes, tardigrades, rotifers and protists were all found at Junction Spur (Table S2). Although most faunal groups found elsewhere in the Transantarctic Mountains were present in the Darwin Mountains region, invertebrate species diversity was found to be low. It is interesting to note that the occurrence of invertebrates at LW19 (likely to be on Isca drift, see below), and Junction Spur sites (Hatherton drift) coincide with samples that were found to be similar in terms of microbial community structure (Figs. S2a,b).

**Figure 3:**
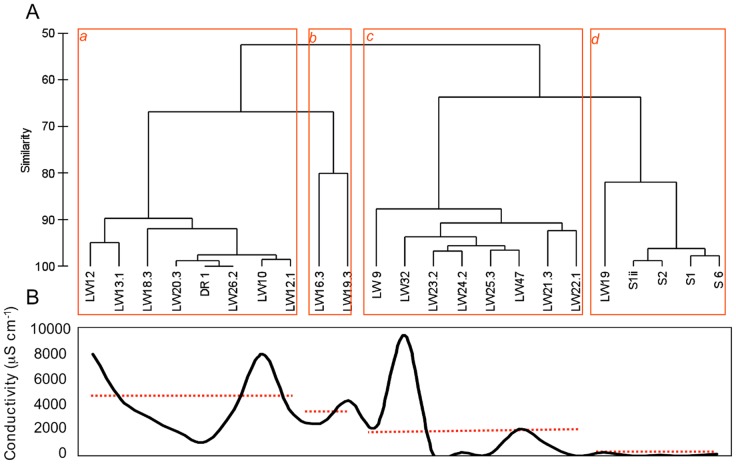
Hierarchical clustering of biota richness (A) and spatial variation of soil conductivity (B). Hierarchical clustering analysis was performed based on group average linking of Euclidean distances calculated for fourth root richness data of bacteria, cyanobacteria, invertebrates, lichens and algae presented at Table S2. Spatial variation of conductivity was plotted for the samples analysed by hierarchical clustering analysis; dashed lines represent mean conductivity values for the samples grouped within each cluster generated.

To identify spatial diversity differences in all groups of organisms (bacteria, cyanobacteria, invertebrates, lichens and algae) within the sampling area of the Darwin Mountains, an HC analysis was performed based on the diversity matrix generated for all groups of soil organisms (using Richness values) (Fig. 3). Results showed that samples grouped in four main clusters (ANOSIM *R* = 0.96, *p*<0.01), with a different complexity with respect to the presence of the group of organisms analysed. Cluster *d* included samples that contained higher diversity of all groups of organisms evaluated (Junction Spur samples and LW19). This group of sampling sites were also more similar in terms of bacterial and cyanobacterial community structure (Figs. S2a,b). Conversely, cluster *a* was composed of less complex samples; only bacteria were found to occur at these sites. At cluster *c* all samples were composed of bacterial and cyanobacterial communities and cluster *b* samples were composed only of bacteria, lichens and, at LW19.3, tardigrades.

### Biological Diversity vs Environmental Controls

When conductivity values were compared with the HC analysis generated for richness data from all group of organisms analysed (Figs. 3a,b), it became clear that samples that support more complex communities (LW19, and all Junction Spur sites; Fig. 3) were associated with lower mean soil conductivity values and thus lower ions concentrations. On the other hand, samples comprising only bacteria generally had the highest mean conductivity values. In other words, bacteria dominated over cyanobacteria, invertebrates, lichens and algae at higher salinities, but bacterial diversity was greater at lower salinities. Thus, the gradient from less to high complexity communities in samples that were included in cluster *a*, *b*, *c* and *d*, respectively, was followed by a general congruent gradient of conductivity (Fig. 3a,b).

**Figure 4:**
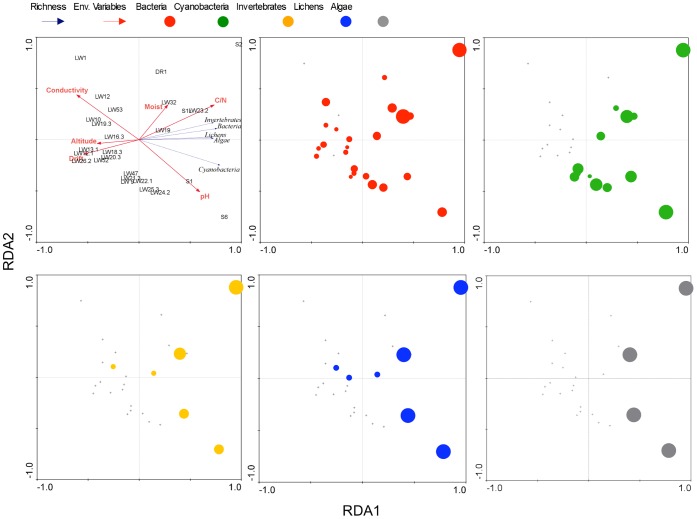
Redundancy analysis ordination (RDA) plot for the biogeochemical/geographic variables and richness of the different groups of organisms analyzed. Environmental variables included in the analysis (Table 1; Table S1) were found to contribute significantly to the explanation of different richness distributions. Richness values for bacteria, cyanobacteria, invertebrates, lichens and algae are represented as circles of diameter scaled linearly to the magnitude of the value.

Correlations between environmental variables and richness (number of different AFLs or species of each group of organisms analysed) were also examined using redundancy analysis (RDA; Fig. 4). From the original environmental variables presented in Table 1, only six contributed significantly to the different richness distributions resolved by the Monte Carlo test of F-ratios (*F* = 5.140 and *p* = 0.002). The first gradient (RDA 1, horizontal) explained 59.2% of the total richness variability and was well correlated with the environmental data (95.2%), suggesting that the data set is governed by a single dominant gradient represented by RDA 1 (horizontal). The RDA projection of the environmental variables revealed that the RDA 1 axis is negatively correlated with altitude, drift and conductivity gradient (representing the concentration of Na, Mg, Ca, Cl and NO_2_+NO_3_) and positively correlated with C/N ratios and the pH gradient (Fig. 4). The correlation matrix generated by RDA analysis confirmed that relationships of all measured environmental variables with the second axis (RDA 2, vertical) were rather weak, with the exception of moisture. The position of the individual richness data showed that the high diversity of all organisms evaluated is closely related to lower conductivity, altitude and drift values (lower drift values represents lower drift ages). The antagonistic relationship between drift ages and diversity is particularly evident for bacteria and cyanobacteria, the more widely distributed organisms analysed in our sampling area (Fig. S3). These results together suggested that the higher diversity and more complex communities were observed in soils on Hatherton and Britannia drifts (except LW19, observed on Isca drifts) assigned by Bockheim et al.^30^ to weathering stage 1 (weakly developed); soils with lower or no coherence and very little total soluble salt content. In Figure 4, the size of the symbols corresponds to the species or AFLs numbers for each group of organisms analyzed. Results showed generally higher diversity of bacteria, cyanobacteria, invertebrates, lichens and algae in samples with lower conductivity (and thus lower values of NO_3_^−^+NO_2_^−^, Cl, Mg, Ca, and Na, which covary with conductivity), higher C/N ratio and pH, and located at lower altitude and in the younger age glacial drift terrain.

## Discussion

Antarctic soil ecosystems are being recognized as ideal environments to test our hypotheses about how soil geochemistry can account for variation in biodiversity and ecosystem function [7], [12], [16]. The extreme and high heterogenic environmental conditions of Antarctic soils and the simplicity of biotic communities facilitate direct assessment to the environmental drivers on distribution and diversity of the main contributors to ecosystem processes. Particularly the remote ice-free regions of the Darwin Mountains characterized by multiple drift sheets and accompanied by a high range of simple multi-trophic diversity, are unique characteristics which make it possible to test/relate the importance of soil geological history in driving biological diversity. In this study we coupled biodiversity at multiple trophic levels, historical landscape change and environmental factors to identify keystone drivers of the presence and distributions of taxa in the Darwin Mountains, continental Antarctica. Our findings revealed that abiotic spatial heterogeneity and geological and glacial history are fundamental to understanding constraints influencing biological distribution in Antarctic soil ecosystems. Our findings illuminate previous research on biota from other Antarctic extreme cold desert environments that suggest a number of drivers, such as source and composition of organic matter, availability of liquid water, and soil salinity [6], [7], [12], [19], [28], [29], [32]. Here, we suggest that carbon content, nutrient availability, and soil water content are secondary driving forces for biotic distribution, but that soil salinity, as a function of drift age, is the keystone driver of presence and distribution of biota in the Darwin Mountains of continental Antarctica.

### Biological Diversity vs Environmental Controls

Our data indicated that bacteria are the more widely distributed organisms in our sampling area. Soils with lower conductivity and higher C/N ratios were found to favour a higher diversity of these microorganisms. Oxygenic phototrophs (cyanobacteria), are major contributors to basic ecosystem processes in the Antarctic Dry Valleys [6] and have shown similar patterns at high-altitude Himalayan arid soils^33^. However, in Antarctica we found that they have a more constrained distribution in the Darwin Mountains, with no detection (below the detection limit) of this group of organisms in 43% of the study sites. This was unexpected due to the great ability of cyanobacteria to grow in undeveloped deglaciated soils and in extremely arid remote regions [6], [33]. Soil water content has been suggested as one of the most important variables in Antarctic soil productivity [6] and in regulating cyanobacterial diversity [16]. However, our data did not show any relationship between moisture and cyanobacterial diversity, which is in agreement with a recent study [18]. Instead, cyanobacterial diversity correlates with soil pH, C/N ratios and soil salt concentration. Greater success of cyanobacteria community development in higher pH soils, has also been recently described in an alpine environment [33]. Distribution of invertebrates, lichens and algae were even more restricted (Table S1). Interestingly, higher diversity of invertebrates, lichens and algae was correlated with the same soil chemical characteristics as the microbial communities: lower soil salt concentrations and high C/N ratios. Thus, our results indicate that soil moisture (range 0.7–11.3%), usually suggested as an important determinant for species presence and distribution in Antarctic environments [6], [19], [21], [34], had no impact on species distribution and diversity in the Darwin Mountains soils. These results indicate that organisms that inhabit the polar desert soils of Darwin Mountains region are adapted to very low moisture conditions (3±2.3%), suggesting that spatial variation of soil water content measured in this region does not limit habitat suitability or richness of the different groups of organisms analysed. Instead conductivity, in particular soil concentrations of Cl, Na, Mg, NO_2_^−^ + NO_3_^−^, was an important variable affecting the distribution of the Darwin Mountain’s biota. Conductivity explained differences in bacterial and cyanobacterial distribution^18^, and was identified as an important factor determining invertebrate habitat suitability in other Antarctic Dry Valleys [16], [22], [23], [35]. Soils from Dry Valleys are often saline due to the lack of precipitation and the accumulation of salts through weathering and atmospheric deposition in the absence of leaching [36]. However, the origin and distribution of salts may depend on several climate and geological variables like soils parental composition, precipitation source, leaching extent, soil temperature, moisture regimes and soil age and weathering episodes [23], [30], [36], [37], [38]. Indeed, studies interpreting water as the limiting resource may be due more to the fact that there has been no previous attempt to link the accumulation of salts with terrain age as we have done here.

### Biological Diversity vs Terrain Age

The ice-free regions alongside Hatherton and Darwin Glaciers, possess multiple drift sheets based on several soil morphological features [30] and cosmogenic ages between late Quaternary to early Holocene [31]. Our sampling sites were distributed between these drifts (Fig. 2, Table 1), which clearly differ in terms of weathering stages and soil properties (Table 1; Table S1 [30]). In this study, the ages of the drift sheets were found to be key factors in driving biological diversity in the Darwin Mountains, with higher diversity and the occurrence of more complex communities (multitrophic) in the younger drifts characterized by weakly developed soils (weathering stage 1), with lower or no coherence and less total soluble salts content. The fact that younger polar soils had less time to accumulate atmospheric ions makes habitat conditions suitable for a higher range of organisms with lower level of osmotic tolerance. While LW19 site was characterised in the oldest drift (Isca) it is located directly below one of the few glacial ice inputs into the region and likely to be ‘flushed’ regularly diluting salts concentrations within the soils. Thus, in this case, hydrologic regime exerts its influence via alterations of soil salinity. Conversely, the extremely high conductivity of the older soils might constrain habitat suitability by increasing osmotic stress. Taken together, we believe that the spatial differences observed in biotic complexity were primarily driven by a gradient of ion concentrations that imposed progressive levels of osmotic stress to organisms limiting their diversity.

### The Influence of Abiotic Drivers on Biological Diversity

Continental Antarctic terrestrial food webs are thought to be among the simplest on the planet, yet we lack a fundamental understanding of the importance of trophic relationships in these systems. It has, however, been previously suggested that the low diversity and abundance of grazing organisms in the Antarctic Dry Valleys and the strong physical and chemical pressures of these ecosystems make abiotic factors more important in controlling the structure of microbial communities than grazing [15], [29], [31]. In this study, the relative diversity of bacterial and cyanobacteria in all of our samples was found to be significantly correlated. These results suggest that both bacteria and cyanobacteria communities respond in the same way to the chemical and physical parameters that ultimately select for suitability of initial community colonization. However, some bacteria seem to possess a high tolerance to these amplitudes of environmental factors leading to a broad dispersion of this group of organisms throughout the study area. While previous studies have shown that bacterial diversity and abundance tend to decrease with increased salinity, these microorganisms have developed extraordinary survival strategies to inhabit extreme saline environments^39^. The fact that cyanobacteria were present only at sites where bacterial assemblages occur, suggest that a pre-development of a bacterial community is necessary to pre-empt the development of cyanobacteria. The macrobiota (invertebrates, lichens and algae) analyzed in this study appear to be scarcely distributed within the Darwin Mountains environments (invertebrates at 6 sites, lichens at 7 sites and algae at 4 sites), which makes it more difficult to statistically analyze the relationships between micro- and macrobiota spatial diversity. While bacteria and cyanobacteria were present at sites with higher levels of salinity, the sites where we observed a higher diversity of macrobiotic communities corresponded directly to areas supporting higher bacterial and cyanobacterial diversity, indicating that the presence of well-developed microbial communities must be a prerequisite for the formation of more complex multi-trophic communities.

Our results indicate that the inferred ecological succession of micro- and macro-biota within the different drifts of the Darwin Mountains region was strongly influenced by site geochemistry, more precisely by the high gradient of conductivity of this region. We hypothesized that the gradient of ions concentration from younger to older exposed soils imposes a degree of osmotic stress to the organisms which drives a succession of complex to more simple communities from younger to older drift terrain, and this trend will dominate the environment in the absence of other environmental constraints. These results open new perspectives concerning patterns of biological succession by relating the occurrence of more complex diverse communities to younger drift soils due to the fact that chemical forces tend to get stronger with drift age limiting natural biological succession patterns to occur in older soils, supporting generally simple communities mainly composed of bacteria.

## Materials and Methods

### Sampling Program and Sites Description

Samples from this study were collected during December 2007 at 30 locations distributed in the Darwin-Hatherton Glacier region of the Darwin Mountains, the second largest ice-free region in the Transantarctic Mountains, located in central East Antarctica (Fig. 1). Most of the sampling sites (25 of 30) were located around Lake Wellman region in southeastern Darwin Mountains, in addition to some samples located on Junction Spur (S1, S1ii, S2, S6) and Dusky Ridge (DR1) (Fig. 1); distance between sampling locations varied between 100 m to 20 km. Sampling strategy used was described in Storey *et al*. [31] and utilized knowledge from Bockheim *et al*. [30]. Our aim was to obtain samples that would be distributed across a range of the various chronological ages of terrain (time since glacial ice receded) from recently exposed to the maximum exposures. At each sampling location five soil samples of around 100 g each were collected to a depth of 10 cm using a clean sterilized stainless steel scoop from within a one metre square (centre and 4 corners) and placed into a bag and thoroughly mixed. The soil was then separated into two separate sterile plastic bags and stored in ice chests. Samples were transported in ice chests to Scott Base (and then to Waikato University, NZ, for micro-faunal analysis) or Crary Laboratory at McMurdo Station, for soil chemistry and macro-faunal analyses, samples for DNA extraction were kept at −80°C until further analysis (see below). All necessary permits were obtained for the fieldwork and collections. Required permits were obtained through Antarctica New Zealand and were undertaken as part of Antarctica New Zealand’s “The Latitudinal Gradient Project”.

### Soil Chemical Analysis

Under a laminar flow hood, we removed rocks in each sample that were *>*4 mm and, sub-sampled soils for chemical and biotic analysis (see below). Soil moisture was measured from 25 g of fresh soil, and percentage of moisture content (% g water per g dry soil) calculated by weight loss after drying in 105°C oven for 24 h [21]. For chemical analysis, soils were aseptically sub-sampled, 2 mm sieved and frozen at −20°C until analysis at Dartmouth College [22], [40]. Soil pH and conductivity were measured in 1∶2 and 1∶5 DI-H_2_O extracts, respectively, using a VWR Scientific 8015 pH probe (West Chester, PH, USA) and Orion 160 conductivity meter (Boston, MA, USA). Conductivity values were corrected for temperature using a standard of 0.01 M KCl solution. Inorganic forms of N (NH_4_^+^, NO_2_^−^ + NO_3_^−^) were measured by extracting 15 g of soil in 50 ml of 2 M KCl for 60 min at 180 rpm. The supernatant was filtered and the filtrate stored at −20°C until analyzed. A similar procedure was used for ortho-phosphates, except that extraction was performed in 0.5 M NaHCO_3_ at pH 8.5. Filtrates were analyzed on a Lachat QuikChem 8500 (Loveland, CO, USA). All soluble ions (Cl, Fl, Br, Li, Na, K, Mg, Ca) were extracted in 50 ml DI-H_2_O using 10 g of soil. The supernatant was filtered to 0.45 µm and frozen at −20°C before being analysed on a Dionex DX-120 IC (Sunnyvale, CA, USA). Total C and N were measured from approximately 60 mg of oven-dried, hand-ground (via sapphire mortar & pestle) soil. A 1-g subsample of the dry ground soil was neutralized with 1 ml 6N HCl and oven dried for measurement of organic C. All samples were kept in a desiccator until analysis on a Carlo Erba Elemental Analyzer (Milan, Italy).

### Invertebrate Analysis

We extracted soil invertebrates from a subsample of fresh soil (100 g) using a modified sugar centrifugation technique [41]. Invertebrates were enumerated and identified using light microscopy (400×) within 48 h following extraction. Mites (*Stereotydeus* sp. and one unknown sp.) were identified to genus and nematodes (*Scottnema lindsayae*) were identified to species. Tardigrades, rotifers, and protists were inumerated but not identified further. Total invertebrate abundance was expressed per kilogram of soil (oven dry weight equivalent).

### Bacterial and Cyanobacterial Analysis

From each sample site six replicates of DNA were extracted, each from 0.6 to 0.8 g of homogenized soil, stored at −80°C, using a modification of the CTAB (bromide-polyvinylpyrrolidone-bmercaptoethanol) extraction protocol [6], [40]. Each of three replicates of extracted DNA was combined and ITS regions, in the rRNA operon, was amplified in duplicate 50 µl volumes containing universal bacterial and cyanobacteria specific primers (Table S3; Invitrogen, Auckland, New Zealand) according to Cardinale et al. [43] and Wood *et al*. [18], respectively. The primers ITSReub and CY-ARISA-F were labelled with the phosphoramidite dye HEX (6-carboxy-1,4-dichloro-2,4,5,7-tetra-chlorofluorescein) and 6-FAM (6-carboxyfluorescein) respectively. PCRs mixtures contained between 10–30 ng of DNA 300 nM of both primers, 200 µM dNTPs (Roche Diagnostics, Auckland, New Zealand), 1x Taq PCR buffer, 1.5 U Platinum Taq DNA polymerase (Invitrogen, Auckland, New Zealand), 2.4 mM MgCl and 0.6 µg bovine serum albumin (Sigma, Auckland, New Zealand). The PCR mixture was held at 94°C for 2 min, followed by 30 cycles of 94°C for 45 s, 55°C for 30 s for bacteria and 50°C for 30 s for cyanobacteria, 72°C for 2 min, and a final extension at 72°C for 7 min. Duplicate PCR products from triplicate total DNA extractions were combined, purified and quantified with a NanoDrop spectrophotometer (Thermo Scientific). Standardized amount of the purified PCR product was mixed with an internal size standard (ROX 1000, Applied Biosystems) and ARISA fragments determined using the MegaBACE system (Amersham Pharmacia Biotech, Auckland, New Zealand) at the University of Waikato Sequencing Facility (Hamilton, New Zealand).

### Identification of Lichens

Identification of clearly assignable lichens was performed in the field and confirmed in the lab based on the morphological characteristics and non-clearly assignable lichens were identified based on molecular analyses. Total DNA was extracted from thallus or apothecia using the DNeasy Plant Mini Kit (Qiagen) according to the manufacturer’s instructions. The internal transcribed spacer region (ITS) of the mycobionts’ nuclear ribosomal DNA was amplified and sequenced with the primers ITS1-F [44] and ITS4 [45] (Table S2) according to the protocol described in Ruprecht et al. [46]. To identify the species the obtained sequences (GU074435 - GU074437, GU170839 - GU170842) were aligned with homologous sequences from the NCBI-Database and with yet unpublished sequences from Antarctic lichens of the herbarium of the University of Salzburg (SZU).

### Statistics

Automated rRNA intergenic spacer analysis fragment lengths (ARISA - AFLs) were analyzed by Genetic Profiler V.2. (GE Healthcare) and the data was further processed by normalizing the peak areas and true peaks identified using previous developed algorithms [47]. AFLs of less than 120 bp for Bacterial and those smaller than 180 bp for cyanobacteria were considered to be too short to be true ITSs and were removed from the analysis. All intergenic spacer fragments lengths (ARISA-AFLs) data were transposed to presence/absence and Hellinger transformed^48^ (vegan package in R2.15) prior statistical analyses. Since ARISA is a PCR-based method it is not correct to use the relative fluorescence of individual peaks as a proxy of relative abundance of each phylotype. Multivariate analysis from all sites was performed using multidimensional scaling (MDS) and hierarchical cluster (HC) based on Bray–Curtis similarities to detect inter-site differences and/or similarities in bacteria and cyanobacteria diversity [49]. Principal components (PCA) and HC analysis were applied to the environmental and biogeochemical variables measured during the monthly sampling program (Table 1; Table S1). The software package PRIMER version 6 [49] was used to perform the latter multivariate statistical analysis. Relationships between richness of all groups of organisms (bacteria, cyanobacteria, invertebrates, lichens and algae) and soil chemistry variables were analyzed with using multivariate ordination tolls. A detrended correspondence analysis (DCA), revealed that the gradient length of the ordination axis was less than 1, thus a linear response model was most applicable [50]. Redundancy analysis (RDA) was therefore selected as the preferred ordination method [50], using the software package CANOCO (version 4.5, Microcomputer Power, Ithaca, NY) [50]. For RDA, richness data of the organisms from the different trophic levels (bacteria, cyanobacteria, invertebrates, lichens and algae) were log (*n*+1) transformed, and the environmental variables were normalized (i.e. adjusted for a mean of 0 and SD of 1). We used a Monte Carlo permutation test to assess the statistical significance of the relationships. In the RDA ordination diagram, the angle and length of the arrow relative to a given axis reveals the extent of correlation between the variable and the canonical axis (environmental gradient). Geographic Information System methods (ArcView GIS v 9.3.1; ESRI, USA) were used for the geographical representation of the sampling sites.

## Supporting Information

Figure S1 **Principal component analysis (PCA) two dimensional plots of the geochemical data presented in** **Table 1** **.** Values of conductivity (**a**), similar graph was obtained for NO_2_^−^ + NO_3_^−^ Cl, Mg, Ca, Na, since these ions drives conductivity values, and C/N ratio (**b**) for each sample site were represented as circles of diameter scaled linearly to the magnitude of the value. PCA1 and PCA2 together explained 85.8% (PCA1–70.3%; PCA2–15.5%) of the total variability seen in the analysis. Clusters generated by hierarchical cluster analysis based on group average linking of Euclidean distances calculated for the same log-transformed geochemical data were project on the PCA plot (Euclidean distance level of 3.4; ANOSIM *R* = 0.95, *p*<0.01) (d); two or three clusters of samples were generated at the Euclidean distance level of 5.2 and 4.2, respectively.
(TIFF)

Figure S2 **Non-metric multidimensional scaling ordination analysis of the cyanobacteria** (**A**) **and bacteria** (**B**) **AFLs.** Analysis was performed by using average linkage of Bray–Curtis similarities using the Hellinger*-*transformed presence*-*absence data as input variables. Stress value = 0.18. Clusters generated by hierarchical cluster analysis based average linkage of Bray–Curtis similarities calculated for the same data were project on the MDS plot, points enclosed by green and red circles cluster at 32% similarity (ANOSIN, *R* = 0.95, *p*<0.01).
(TIFF)

Figure S3 **Relation between the variability of the number of bacteria (a) and cyanobacteria (b) ARISA-AFLs and drift ages of each sampling site.**
(TIFF)

Table S1 **Chemical properties of soil samples from all sampling sites (n.a. = not available).**
(DOC)

Table S2 **Biological characterization of the soils samples from all sampling sites (n.a. = not available, –  =  not found).**
(DOC)

Table S3 Oligonucleotide probes used in this study.
(DOC)

## References

[1] Stevens MI, Greenslade P, Hogg ID, Sunnucks P (2006) Examining Southern Hemisphere springtails: could any have survived glaciation of Antarctica? Mol Biol Evol 23: 874–882. 16326749 10.1093/molbev/msj073

[2] Convey P, Stevens MI (2007) Antarctic Biodiversity. Science 317: 1877–1878. 17901323 10.1126/science.1147261

[3] Convey P, Gibson JAE, Hillenbrand C-D, Hodgson DA, Pugh PJA, et al (2008) Antarctic terrestrial life – challenging the history of the frozen continent? Biol Rev 83: 103–117. 18429764 10.1111/j.1469-185X.2008.00034.x

[4] Convey P, Stevens MI, Hodgson DA, Hillenbrand C-D, Clarke A, et al (2009) Antarctic terrestrial life – ancient evolutionary persistence or recent colonisation? Quat Sci Rev 28: 3035–3048.

[5] Cowan D, Ah Tow L (2004) Endangered Antarctic microbial communities. Ann Rev Microbiol 58: 649–690. 15487951 10.1146/annurev.micro.57.030502.090811

[6] Barrett JE, Virginia RA, Wall DH, Cary SC, Adams BJ, et al (2006a) Co-variation in soil biodiversity and biogeochemistry in Northern and Southern Victoria Land, Antarctica. Antarctic Science 18: 535–548.

[7] Cary SC, McDonald IR, Barrett JE, Cowan DA (2010) On the rocks: the microbiology of Antarctic Dry Valley soils. Nat Rev Microbiol 8: 129–138. 20075927 10.1038/nrmicro2281

[8] Fernandez-Carazo R, Hodgson DA, Convey P, Wilmotte A (2011) Low cyanobacterial diversity in biotopes of theTransantarctic Mountains and Shackleton Range (80–82°S), Antarctica. FEMS Microbiol Ecol 77: 503–517. 21592144 10.1111/j.1574-6941.2011.01132.x

[9] Peeters K, Hodgson DA, Convey P, Willems A (2011) Culturable Diversity of Heterotrophic Bacteria in Forlidas Pond (Pensacola Mountains) and Lundström Lake (Shackleton Range), Antárctica. Microb Ecol 62: 399–413. 21424822 10.1007/s00248-011-9842-7

[10] Martiny JBH, Bohannan BJM, Brown JH, Colwell RK, Fuhrman JA, et al (2006) Microbial biogeography: putting organisms on the map. Nat Rev Microbiol 4: 102–112. 16415926 10.1038/nrmicro1341

[11] Oline DK (2006) Phylogenetic comparisons of bacterial communities from serpentine and nonserpentine soils. Appl Environ Microbiol 72: 6065–6071. 10.1128/AEM.00690-06 PMC1636195 16950906

[12] Adams BJ, Bardgett RD, Ayres E, Wall DH, Aislabie J, et al (2006) Diversity and distribution of Victoria Land biota. Soil Biol Biochem. 38: 3003–3018.

[13] Peat HJ, Clarke A, Convey P (2007) Diversity and biogeography of the Antarctic flora. Journal Biogeography 34: 132–146.

[14] Cameron R, Morelli FA, Johnson RM (1972) Bacterial species in soil and air of the Antarctic continent. Antarctic Journal 7: 187–198.

[15] Hogg ID, Cary SC, Convey P, Newsham KK, O’Donnell AG, et al (2006) Biotic interactions in Antarctic terrestrial ecosystems: Are they a factor? Soil Biol Biochem 38: 3035–3040.

[16] Barrett JE, Virginia RA, Hopkins DW, Aislabie J, Bargagli R, et al (2006b) Terrestrial ecosystem processes of Victoria Land, Antarctica. Soil Biol Biochem 38: 3019–3034.

[17] Smith JJ, Ah Tow L, Stafford W, Cary C, Cowan DA (2006) Bacterial diversity in three different Antarctic cold desert mineral soils. Microb Ecol 51: 413–421. 16596438 10.1007/s00248-006-9022-3

[18] Wood S, Rueckert A, Cowan D, Cary C (2008) Sources of edaphic cyanobacterial diversity in the Dry Valleys of Eastern Antarctica. ISME J 2: 308–320. 18239611 10.1038/ismej.2007.104

[19] Niederberger TD, McDonald IR, Hacker AL, Soo RM, Barrett JE, et al (2008) Microbial community composition in soils of Northern Victoria Land, Antarctica. Environ Microbiol 10: 713–1724. 10.1111/j.1462-2920.2008.01593.x 18373679

[20] Smith JL, Barrett JE, Tusnady G, Rejto L, Cary SC (2010) Resolving environmental drivers of microbial community structure in Antarctic soils. Antarctic Science 22: 673–680.

[21] Barrett J, Wall D, Virginia R, Parsons A, Powers L, et al (2004) Biogeochemical parameters and constraints on the structure of soil biodiversity. Ecology 85: 3105–3118.

[22] Nkem JN, Wall DH, Virginia RA, Barrett JE, Broos EJ, et al. (2005) Wind dispersal of soil invertebrates in the McMurdo Dry Valleys, Antarctica. Polar Biol 29, 346–352.

[23] Poage MA, Barrett JE, Virginia RA, Wall DH (2008) The influence of soil geochemistry on nematode distribution, McMurdo Dry Valleys, Antarctica. Arc Antar Alp Res 40: 119–128.

[24] Bahl J, Lau MCY, Smith GJD, Vijaykrishna D, Cary SC, et al.. (2011) Ancient origins determine global biogeography of hot and cold desert cyanobacteria. Nat Commun, DOI: 10.1038/ncomms1167. 10.1038/ncomms1167 PMC3105302 21266963

[25] Bockheim JG (1997) Properties and classification of cold desert soils from Antarctica. Soil Sci Soc Am Jour 61: 224–231.

[26] Burkins M, Virginia R, Chamberlain P, Wall D (2000) Origin and distribution of soil organic matter in Taylor Valley, Antarctica Ecology. 81: 2377–2391.

[27] Elberling B, Gregorich EG, Hopkins DW, Sparrow AD, Novis P, et al (2006) Distribution and dynamics of soil organic matter in an Antarctic dry valley. Soil Biol Biochem 38: 3095–3106. 10.1098/rspb.2006.3595 PMC1635502 17015369

[28] Stevens MI, Frati F, McGaughran A, Spinsanti G, Hogg ID (2007) Phylogeographic structure suggests multiple glacial refugia in northern Victoria Land for the endemic Antarctic springtail Desoria Klovstadi (Collembola, Isotomidae). Zool Scripta 36: 201–212.

[29] Takacs-Vesbach C, Zeglin L, Barrett JE, Gooseff MN, Priscu JC (2010) Factors promoting microbial diversity in the McMurdo Dry Valleys, Antarctica. In: Life in Antarctic Deserts and other cold dry environments: Astrobiological Analogs, Doran P, Lyons WB, McKnight DM (ed), pp221–257. Cambridge University Press.

[30] Bockheim JG, Wilson SC, Denton GH, Andersen BG, Stuiver M (1989) Late Quaternary Ice-Surface Fluctuations of Hatherton Glacier Transantarctic Mountains. Quat Res 31: 229–254.

[31] Storey BC, Fink D, Hood D, Joy K, Shulmeister J, et al (2010) Cosmogenic nuclide exposure age constraints on the glacial history and implications on biogeography of the Lake Wellman area, Darwin Mountains, Antarctica. Antarctic Science 22: 603–618.

[32] Adams B, Wall D, Gozel U, Dillman A, Chaston J, et al (2007) The southernmost worm, Scottnema lindsayae (Nematoda): diversity, dispersal and ecological stability. Polar Biol 30: 809–815.

[33] Řeháková K, Chlumská Z, Doležal J (2012) Soil cyanobacterial and microalgal diversity in dry mountains of Ladakh, NW Himalaya, as related to site, altitude, and vegetation. Microb Ecol 62: 337–346. 10.1007/s00248-011-9878-8 21643700

[34] Porazinska DL, Wall DH, Virginia RA (2002) Population age structure of nematodes in the Antarctic Dry Valleys: perspectives on time, space, and habitat suitability. Arc Antar Alp Res 34: 159–168.

[35] Treonis A, Wall D, Virginia R (1999) Invertebrate biodiversity in Antarctic Dry Valley soils and sediments. Ecosystems 2: 482–492.

[36] Campbell I, Claridge G (1987) Antarctica: Soils, weathering processes and environment. United States: Elsevier Science Pub. Co. Inc., New York, NY.

[37] Bockheim JG (2002) Landform and Soil Development in the McMurdo Dry Valleys, Antarctica: A Regional Synthesis. Arc. Antar Alp Res 34: 308–317.

[38] Ugolini FC, Bockheim JG (2008) Antarctic soils and soil formation in a changing environment: A review. Geoderma 144: 1–8.

[39] Dong H (2008) Microbial life in Extreme Environments: Linking Geological and Microbiological Processes. In Dilek, et al. (eds) Links between geological processes, microbial activities & evolution of life, Springer.

[40] Barrett J, Virginia R, Wall D (2002) Trends in resin and KCl-extractable soil nitrogen across landscape gradients in Taylor Valley, Antarctica. Ecosystems 5, 289–299.

[41] Freckman DW, Virginia RA (1993) Extraction of nematodes from Dry Valley Antarctic soils. Polar Biol 13: 483–487.

[42] Coyne KJ, Hutchins DA, Hare CE, Cary SC (2001) Assessing temporal and spatial variability in Pfiesteria piscicida distributions using molecular probing techniques. Aquat Microb Ecol 24: 275–285.

[43] Cardinale M, Brusetti L, Quatrini P, Borin S, Puglia AM, et al (2004) Comparison of Different Primer Sets for Use in Automated Ribosomal Intergenic Spacer Analysis of Complex Bacterial Communities. Appl Environ Microbiol 70: 6147–6156. 15466561 10.1128/AEM.70.10.6147-6156.2004 PMC522057

[44] Gardes M, Bruns TD (1993) ITS primers with enhanced specifity for basidiomycetes – application to the identification of mycorrhizae and rusts. Mol Ecol 2: 113–118. 8180733 10.1111/j.1365-294x.1993.tb00005.x

[46] Ruprecht U, Lumbsch HT, Brunauer G, Green TGA, Türk R (2010) Diversity of *Lecidea* (Lecideaceae, Ascomycota) species revealed by molecular data and morphological characters. Antarctic Science 22: 727–741.

[47] Abdo Z, Schüette UME, Bent SJ, Williams CJ, Forney LJ, Joyce P (2006) Statistical methods for characterizing diversity of microbial communities by analysis of terminal restriction fragment length polymorphisms of 16S rRNA genes. Environ Microbiol 5: 929–938. 10.1111/j.1462-2920.2005.00959.x 16623749

[48] Legendre P, Gallagher ED (2001) Ecologically meaningful transformations for ordination of species data. Oecologia 129: 271–280. 28547606 10.1007/s004420100716

[49] Clarke KR, Warwick RM (1994) Change in marine communities: an approach to statistical analysis and interpretation. Plymouth Marine Laboratory, Plymouth, UK.

[50] ter Braak CJF, Smilauer P (2002) CANOCO reference manual and CanoDraw for Windows user’s guide: software for Canonical Community Ordination (version 4.5), 500 pp. Microcomputer Power, Ithaca, NY, USA.

