# Supplementary figures and images for: At Limits of Life: Multidisciplinary Insights Reveal Environmental Constraints on Biotic Diversity in Continental Antarctica
Source: PLoS One. 2012 Sep 19;7(9):e44578. doi: 10.1371/journal.pone.0044578 (PMC3446939; doi:10.1371/journal.pone.0044578)

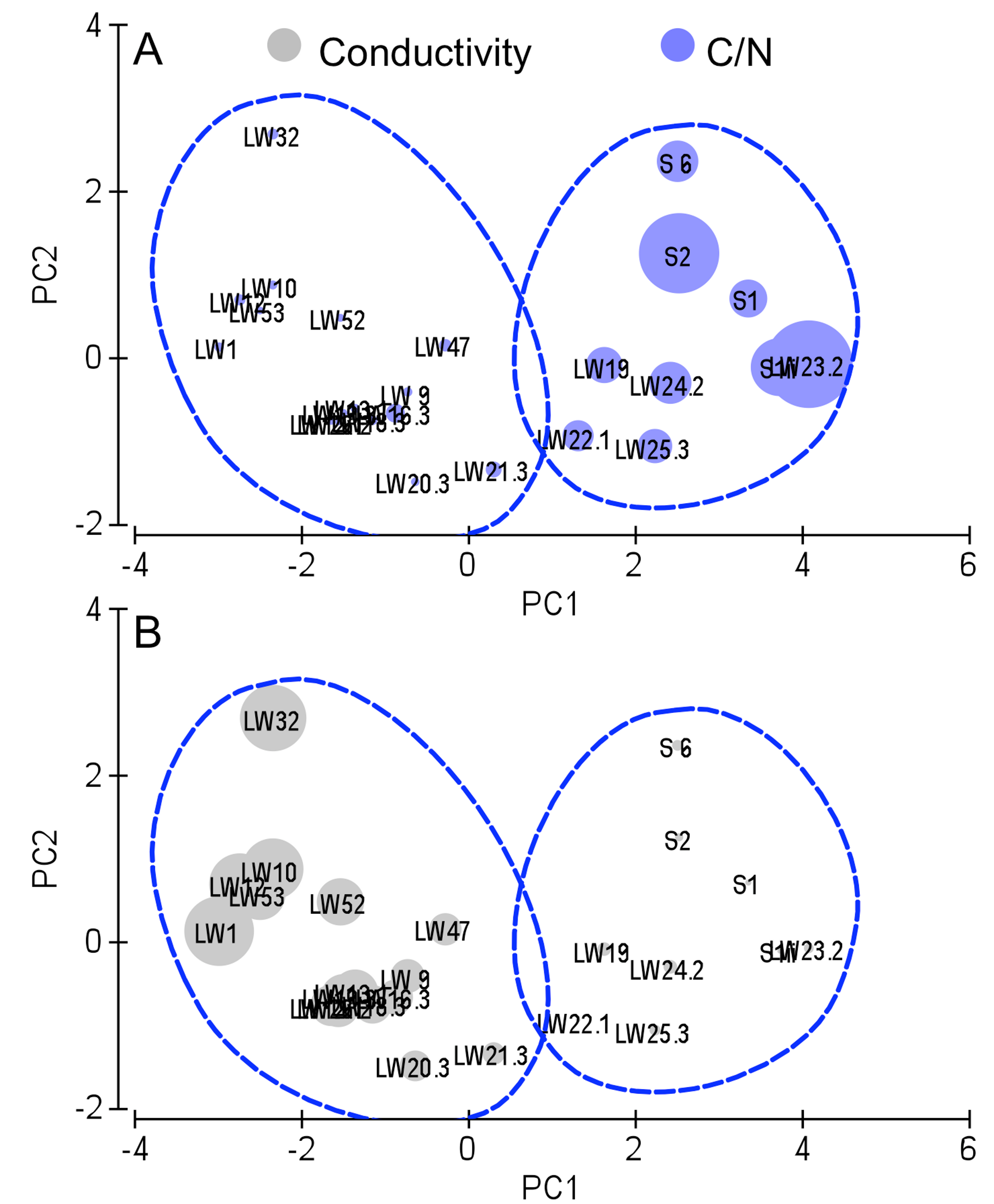

Supplement: Figure S1 — Principal component analysis (PCA) two dimensional plots of the geochemical data presented in Table 1 . Values of conductivity (a), similar graph was obtained for NO2 − + NO3 − Cl, Mg, Ca, Na, since these ions drives conductivity values, and C/N ratio (b) for each sample site were represented as circles of diameter scaled linearly to the magnitude of the value. PCA1 and PCA2 together explained 85.8% (PCA1–70.3%; PCA2–15.5%) of the total variability seen in the analysis. Clusters generated by hierarchical cluster analysis based on group average linking of Euclidean distances calculated for the same log-transformed geochemical data were project on the PCA plot (Euclidean distance level of 3.4; ANOSIM R = 0.95, p<0.01) (d); two or three clusters of samples were generated at the Euclidean distance level of 5.2 and 4.2, respectively. (TIFF) [file pone.0044578.s001.tiff]

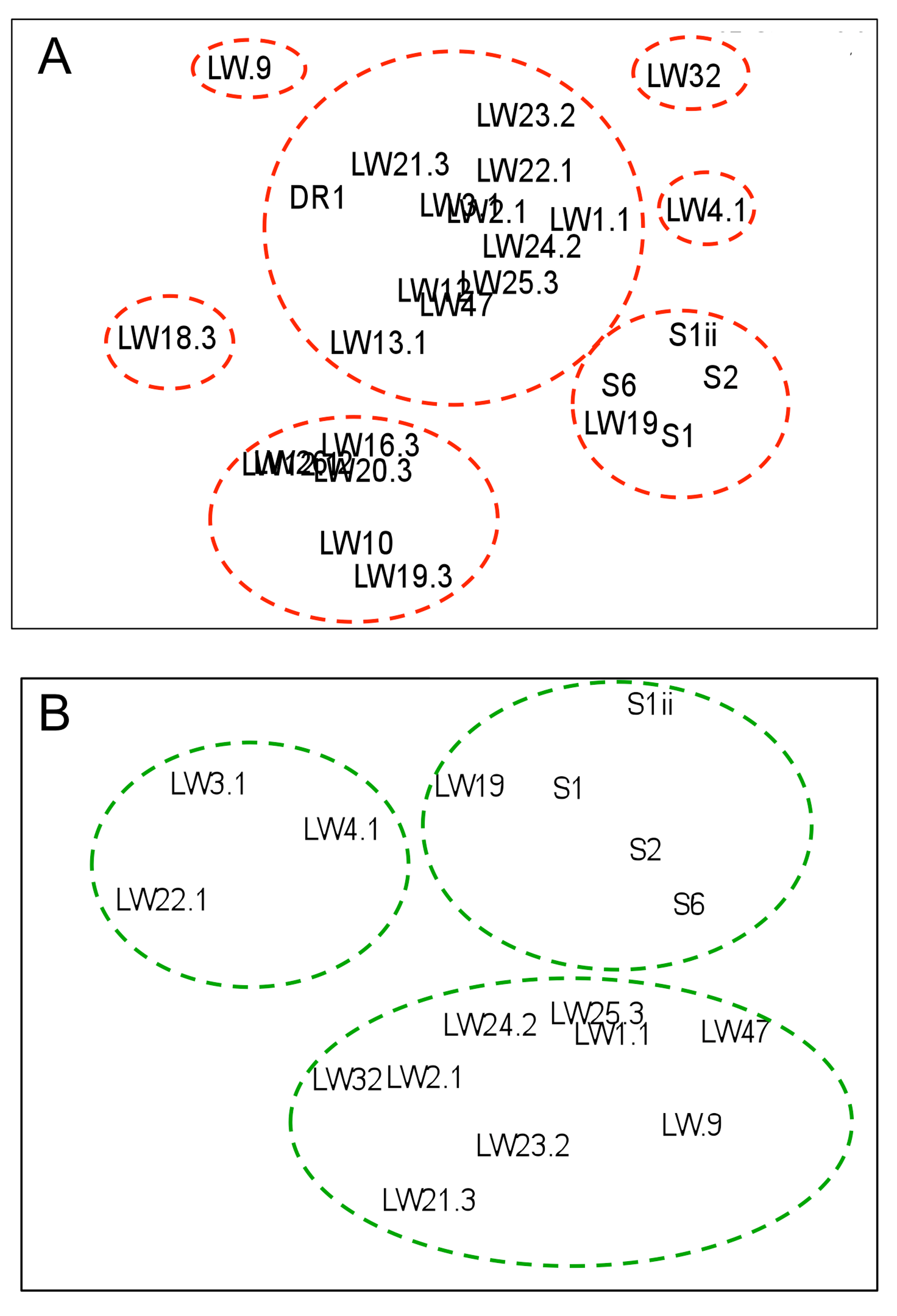

Supplement: Figure S2 — Non-metric multidimensional scaling ordination analysis of the cyanobacteria (A) and bacteria (B) AFLs. Analysis was performed by using average linkage of Bray–Curtis similarities using the Hellinger-transformed presence-absence data as input variables. Stress value = 0.18. Clusters generated by hierarchical cluster analysis based average linkage of Bray–Curtis similarities calculated for the same data were project on the MDS plot, points enclosed by green and red circles cluster at 32% similarity (ANOSIN, R = 0.95, p<0.01). (TIFF) [file pone.0044578.s002.tiff]

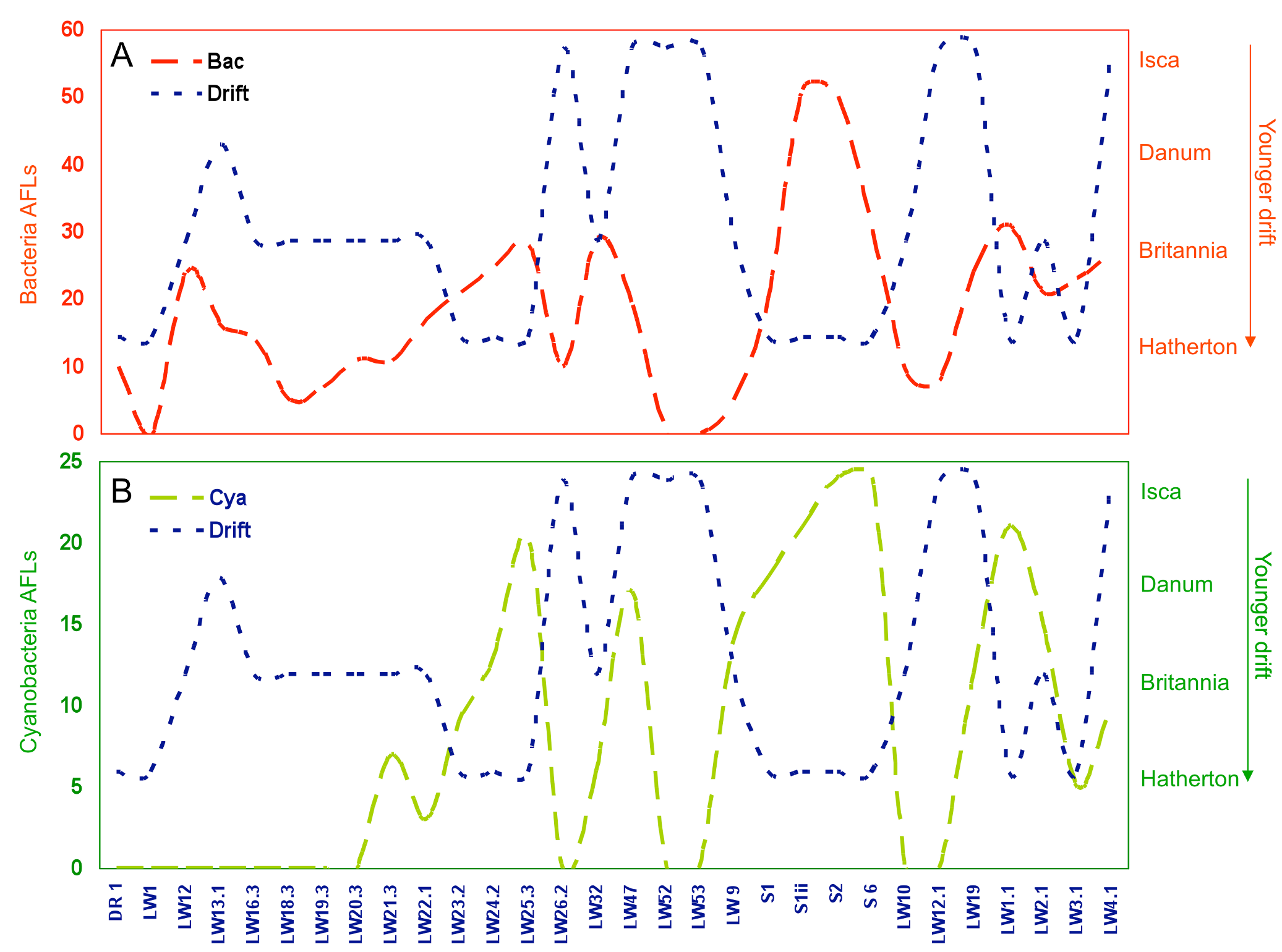

Supplement: Figure S3 — Relation between the variability of the number of bacteria (a) and cyanobacteria (b) ARISA-AFLs and drift ages of each sampling site. (TIFF) [file pone.0044578.s003.tif]
