# Supplementary material for: At Limits of Life: Multidisciplinary Insights Reveal Environmental Constraints on Biotic Diversity in Continental Antarctica
Source: PLoS One. 2012 Sep 19;7(9):e44578. doi: 10.1371/journal.pone.0044578 (PMC3446939; doi:10.1371/journal.pone.0044578)
Supplement: Table S1 — Chemical properties of soil samples from all sampling sites (n.a. = not available). (DOC) [file pone.0044578.s004.doc]

| **ID** | **NH_4_** | **NO_3_^-^+ NO_2_^-^** | **PO_4_** | **CL** | **FI** | **Br** | **Li** | **Na** | **K** | **Mg** | **Ca** |
| --- | --- | --- | --- | --- | --- | --- | --- | --- | --- | --- | --- |
|  | **μg / g soil** | | | | | | | | | | |
| **LW23.2** | 0.21 | 4.81 | 0.33 | 7.95 | 3.02 | 0 | 0 | 90.04 | 0 | 0 | 54.21 |
| **LW25.3** | 1.17 | 27.78 | 0.00 | 67.61 | 0 | 0 | 0 | 44.90 | 0 | 17.69 | 100.10 |
| **LW1.1** | n.a. | n.a. | n.a. | n.a. | n.a. | n.a. | n.a. | n.a. | n.a. | n.a. | n.a. |
| **LW2.1** | n.a. | n.a. | n.a. | n.a. | n.a. | n.a. | n.a. | n.a. | n.a. | n.a. | n.a. |
| **LW1** | 2.67 | 3535.38 | 0.14 | 9997.78 | 2.44 | 0 | 0 | 3721.75 | 26.14 | 1539.05 | 5345.77 |
| **LW24.2** | 0.21 | 20.85 | 0.31 | 97.55 | 3.89 | 0 | 0 | 163.34 | 2.02 | 26.29 | 129.59 |
| **LW22.1** | 0.52 | 273.96 | 0.14 | 1046.31 | 0 | 0 | 0 | 0 | 9.59 | 174.87 | 576.77 |
| **LW10** | n.a. | n.a. | 0.00 | n.a. | n.a. | n.a. | n.a. | n.a. | n.a. | n.a. | n.a. |
| **LW9** | 0.41 | 708.00 | 0.03 | 1736.19 | 0 | 0 | 0 | 757.90 | 18.46 | 378.54 | 844.02 |
| **LW16.3** | 1.55 | 645.28 | n.a. | n.a. | n.a. | n.a. | n.a. | n.a. | n.a. | n.a. | n.a. |
| **LW3.1** | n.a. | n.a. | n.a. | n.a. | n.a. | n.a. | n.a. | n.a. | n.a. | n.a. | n.a. |
| **LW12** | 1.86 | 2918.48 | 0.00 | 7671.75 | 0 | 0 | 0 | 33.68.61 | 97.29 | 2051.38 | 2944.30 |
| **LW21.3** | 0.1 | 261.26 | 0.12 | 513.73 | 0 | 0 | 0 | 212.24 | 5.40 | 109.13 | 603.67 |
| **LW32** | 1.99 | 2470.24 | 0.00 | 9719.29 | 0 | 0 | 4.15 | 9231.83 | 58.01 | 1539.84 | 5877.14 |
| **LW20.3** | 0.47 | 728.5 | 0.52 | 637.95 | 0 | 0 | 0 | 211.37 | 35.09 | 266.49 | 2074.69 |
| **LW19.3** | 0.58 | 1486.15 | 0.21 | 2028.29 | 0 | 0 | 0 | 1702.17 | 20.79 | 541.44 | 4341.79 |
| **LW18.3** | 0.98 | 1257.29 | 0.81 | 1916.51 | 0 | 0 | 0 | 835.19 | 11.17 | 759.16 | 1702.44 |
| **LW13.1** | 0.49 | 1162.94 | 0.54 | 2365.85 | 0 | 0 | 0 | 1617.89 | 28.95 | 357.14 | 380.04 |
| **LW53** | 3.25 | 2829.71 | 2.24 | 2992.55 | 0 | 0 | 0 | 3037.68 | 37.03 | 974.18 | 2768.19 |
| **LW52** | 0.08 | 1518.67 | 0.32 | 2712.99 | 0 | 0 | 0 | 3358.25 | 8.54 | 674.16 | 1847.53 |
| **LW12.1** | n.a. | n.a. | 0.58 | n.a. | n.a. | n.a. | n.a. | n.a. | n.a. | n.a. | n.a. |
| **LW26.2** | 0.04 | 1639.68 | 0.75 | n.a. | n.a. | n.a. | n.a. | n.a. | n.a. | n.a. | n.a. |
| **LW47** | 0.67 | 427.13 | 0.42 | 490.07 | 0 | 0 | 4.09 | 987.32 | 21.90 | 337.60 | 3256.69 |
| **LW19** | n.a. | n.a. | n.a. | n.a. | n.a. | n.a. | n.a. | n.a. | n.a. | n.a. | n.a. |
| **LW4.1** | n.a. | n.a. | n.a. | n.a. | n.a. | n.a. | n.a. | n.a. | n.a. | n.a. | n.a. |
| **S1** | 0.45 | 3.05 | 0.92 | 6.35 | 0 | 0 | 0 | 7.80 | 0 | 5.75 | 11.30 |
| **S1ii** | 0.47 | 0.55 | 0.60 | n.a. | n.a. | n.a. | n.a. | n.a. | n.a. | n.a. | n.a. |
| **S2** | 0.34 | 19.77 | 0.37 | 49.69 | 0 | 0 | 0 | 37.22 | 0 | 7.03 | 14.29 |
| **S6** | 0.94 | 26.58 | 1.01 | 76.56 | 0 | 0 | 0 | 52.36 | 3.47 | 42.66 | 129.62 |
| **DR1** | 60.49 | 193.75 | 3.1 | 407.95 | 5.83 | 0 | 0.23 | 436.35 | 21.97 | 209.92 | 491.69 |
