# Supplementary material for: At Limits of Life: Multidisciplinary Insights Reveal Environmental Constraints on Biotic Diversity in Continental Antarctica
Source: PLoS One. 2012 Sep 19;7(9):e44578. doi: 10.1371/journal.pone.0044578 (PMC3446939; doi:10.1371/journal.pone.0044578)
Supplement: Table S2 — Biological characterization of the soils samples from all sampling sites (n.a. = not available, – = not found). (DOC) [file pone.0044578.s005.doc]

| **Sample** | **Average AFLs in sample** | | **Invertebrates** | | **Lichens** | **Algae** |
| --- | --- | --- | --- | --- | --- | --- |
|  | **Bacterial** | **Cyanobacterial** | **Nº of individuals**  **(Kg ^-1^ soil)** | **Groups Identified** |  |  |
| **LW23.2** | 21 | 9 | --- | --- | --- | --- |
| **LW25.3** | 28 | 20 | --- | --- | --- | --- |
| **LW1.1** | 31 | 21 | n.a | n.a | n.a | n.a |
| **LW2.1** | 21 | 15 | n.a | n.a | n.a | n.a |
| **LW1** | --- | --- | --- | --- | --- | --- |
| **LW24.2** | 25 | 13 | --- | --- | --- | --- |
| **LW22.1** | 17 | 3 | --- | --- | --- | --- |
| **LW10** | 9 | --- | --- | --- | --- | --- |
| **LW9** | 5 | 14 | --- | --- | *---* | --- |
| **LW16.3** | 14 | --- | --- | --- | *Lecidea cancriformis* | --- |
| **LW3.1** | 23 | 5 | n.a | n.a | n.a | n.a |
| **LW12** | 24 | --- | --- | --- | --- | --- |
| **LW21.3** | 11 | 7 | --- | --- | --- | --- |
| **LW32** | 29 | 6 | --- | --- | --- | --- |
| **LW20.2** | 11 | --- | --- | --- | --- | --- |
| **LW19.3** | 7 | --- | 20 | tardigrades | *Lecidea cancriformis* | --- |
| **LW18.3** | 5 | --- | --- | --- | --- | --- |
| **LW13.1** | 16 | --- | --- | --- | --- | --- |
| **LW52** | --- | --- | --- | --- | --- | --- |
| **LW53** | --- | --- | --- | --- | --- | --- |
| **LW12.1** | 8 | --- | --- | --- | --- | --- |
| **LW26.2** | 10 | --- | --- | --- | --- | --- |
| **LW47** | 19 | 17 | --- | --- | --- | --- |
| **LW19** | 24 | 12 | 11179 | rotifers | *Lecidea cancriformis* | --- |
| **LW4.1** | 27 | 10 | n.a | n.a | n.a | n.a |
| **S1** | 20 | 18 | 7266 | tardigrades/rotifers/nematodes | *Lecidea cancriformis*/*Lecanora fuscobrunnea*/*Buellia frigida*/  *Acarospora gwynii* | Chlorophytan / Xanthophycean |
| **S1ii** | 51 | 21 | 10251 | tardigrades/rotifers/nematodes/  protists | *Lecidea cancriformis*/*Lecanora fuscobrunnea*/*Buellia frigida*/  *Acarospora gwynii* | Chlorophytan / Xanthophycean |
| **S2** | 50 | 24 | 692 | tardigrades/rotifers/nematodes/  protists/mites | *Lecidea cancriformis*/*Lecanora fuscobrunnea*/*Buellia frigida*/  *Acarospora gwynii* | Chlorophytan / Xanthophycean |
| **S6** | 30 | 24 | 13676 | tardigrades/rotifers/ nematodes | *Lecidea cancriformis*/*Lecanora fuscobrunnea*/*Buellia frigida*/  *Acarospora gwynii* | Chlorophytan / Xanthophycean |
| **DR1** | 10 | --- | --- | --- | --- | ancient algae |
