# Supplementary material for: At Limits of Life: Multidisciplinary Insights Reveal Environmental Constraints on Biotic Diversity in Continental Antarctica
Source: PLoS One. 2012 Sep 19;7(9):e44578. doi: 10.1371/journal.pone.0044578 (PMC3446939; doi:10.1371/journal.pone.0044578)
Supplement: Table S3 — Oligonucleotide probes used in this study. (DOC) [file pone.0044578.s006.doc]

| Target group | Primers | Sequence (5' – 3') | Reference |
| --- | --- | --- | --- |
| Bacteria | ITSF | GTCGTAACAAGGTAGCCGTA | Cardinale et al. 2004  Cardinale et al. 2004 |
|  | ITSReub | HEX-GCCAAGGCATCCACC |  |
| Cyanobacteria | CY-ARISA-F | FAM-GYCAYRCCCGAAGTCRTTAC | Wood et al. 2008  Taton et al. 2003 |
|  | 23S30R | CHTCGCCTCTGTGTGCCWAGGT |  |
| Lichens | ITS1-F | cttggtcatttagaggaagtaa | Gardes and Bruns 1993  White et al. 1990 |
|  | ITS4 | tcctccgcttattgatatgc |  |
